# COVID-19 Prevention Behaviours and Vaccine Acceptability, and Their Association with a Behaviour Change Campaign in Somalia: Analysis of a Longitudinal Cohort

**DOI:** 10.3390/vaccines11050972

**Published:** 2023-05-11

**Authors:** Andrew Seal, Mohamed Jelle, Mohamed Yusuf Hassan, Dek Abdi Farah, Faith Mueni Musili, Janet Micheni, George Samuel Asol, Meena Bhandari, Balint Nemeth

**Affiliations:** 1 UCL Institute for Global Health, London WC1N 1EH, UK; 2 Evidence for Change (e4c), Nairobi 00100, Kenya; 3 Norwegian Refugee Council, Nairobi 00100, Kenya; 4 NORCAP/Norwegian Refugee Council, 0152 Oslo, Norway

**Keywords:** COVID-19, Somalia, SBCC, vaccine hesitancy

## Abstract

Somalia experienced its first wave of COVID-19 infections in March 2020 and has experienced fluctuating infection levels since. Longitudinal data on suspected cases of COVID-19, attitudes, and behaviours were collected by telephone interviews of cash-transfer programme beneficiaries from June 2020–April 2021. A multi-media Social and Behaviour Change Communication (SBCC) campaign was designed and implemented from February 2021 to May 2021. Between the end of the first wave and the onset of the second the perceived threat from COVID-19 increased, with the proportion of respondents viewing it as a major threat increasing from 46% to 70% (*p* = 0.021). Use of face coverings increased by 24% (*p* < 0.001) and hand shaking and hugging for social greeting decreased, with 17% and 23% more people abstaining from these practices (*p* = 0.001). A combined preventative behaviour score (PB-Score) increased by 1.3 points (*p* < 0.0001) with a higher score in female respondents (*p* < 0.0001). During wave 2, vaccine acceptance was reported by 69.9% (95% CI 64.9, 74.5), overall. Acceptance decreased with increasing age (*p* = 0.009) and was higher in males (75.5%) than females (67.0%) (*p* = 0.015). Awareness of the SBCC campaign was widespread with each of the 3 key campaign slogans having been heard by at least 67% of respondents. Awareness of 2 specific campaign slogans was independently associated with an increased use of face coverings (aOR 2.31; *p* < 0.0001) and vaccine acceptance (aOR 2.36; *p* < 0.0001). Respondents reported receiving information on the pandemic from a wide range of sources with mobile phones and radio the most common. Trust in different sources ranged widely.

## 1. Introduction

Non-pharmaceutical interventions (NPI) and vaccination have been widely used to control the spread of COVID-19 and reduce its public health impact [1]. Public knowledge and attitudes are key determinants of the effectiveness of these interventions and a lack of adherence to protective behaviours and vaccine hesitancy have been critical blocks to controlling the pandemic [2,3]. The determinants of these attitudes and behaviours are complex and context specific [4,5,6,7,8]. Intention to be vaccinated against COVID-19 was found to be higher in low- and middle-income countries (LMIC) compared to the USA or Russia but there was considerable variation between countries, and within countries according to sex, while age and educational achievement were also important [9,10]. Further evidence on the prevalence and determinants of COVID-19 vaccine hesitancy and adherence to preventative behaviours has continued to accumulate during the course of the pandemic [11,12,13].

Vaccine hesitancy in Africa has been a major concern and concerted national and international actions to reduce it have been called for [14,15,16,17]. It is thought that acceptability and adherence may be particularly low in conflict-affected countries such as Somalia [18]. Health promotion programmes are more likely to be effective if based on in-depth knowledge of the target population’s knowledge and perceptions, and how those are formed and shaped, as well as being based on a theoretical underpinning [19,20]. However, during the COVID-19 pandemic, there was reduced access to some of the populations most in need, leading to less reliable information on which to base public health decision making.

Data from the government in Mogadishu indicates that the first confirmed case of COVID-19 was found in Somalia on 16 March 2020 [21]. As of 22 January 2022, there had been 26,067 laboratory confirmed cases and 1332 deaths, although these numbers are likely to significantly underestimate the true burden due to low levels of testing. The epidemic curve has fluctuated with repeated waves of infection and increases in crude and cause-specific mortality have been reported [22,23].

Reports have indicated that NPI related behaviours in the initial stages of the outbreak in Somalia were poor, despite efforts by the government in Mogadishu to impose restrictions, and adherence decreased with time [24,25]. With the onset of the second wave of SARS-CoV-2 infections, a UNICEF funded national SBCC campaign was designed and implemented to encourage face coverings, social distancing, hand hygiene, and vaccine acceptance.

This paper describes perceptions and self-reported behaviours from a population cohort participating in a long-term cash transfer programme within regions across the breadth of Somalia, and compares how these changed between the end of the first wave of infections and the start of the second. We also describe the design and coverage of a national SBCC campaign and its association with preventative behaviours and vaccine acceptance. This paper is the first to report an analysis of longitudinal cohort data from Somalia and document the association between a large scale, national, SBBC programme and changes in preventative behaviours and vaccine hesitancy.

## 2. Methods

### 2.1. Setting

Somalia lies in the Horn of Africa and comprises a number of semi-independent states with a central Federal Government in Mogadishu, and the self-declared independent state of Somaliland [26]. The main language is Somali but several other regional languages and dialects, including Maay and Coastal Somali, are also spoken. There is an ongoing conflict between government forces and the non-state armed group, Al-Shabaab. The design and implementation of health promotion campaigns and public health monitoring systems takes place within this complex environment [26].

### 2.2. Study Design

**Figure 1:**
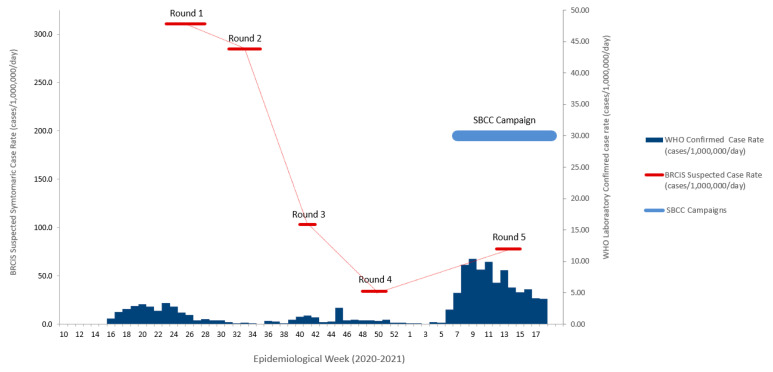
Timing of the BRCiS data collection rounds and the Wave 2 *i*MaskUp SBCC campaign. The horizontal bars indicate the periods for each round of data collection. In the first 2 rounds a one-month recall period was used, contiguous recall periods were used in rounds 3 and 4, and a 3 month recall period (January–March) was used in round 5.

We used a longitudinal household cohort to analyse changes in behaviours and attitudes before and during the second large wave of COVID-19 infections in Somalia, and undertook a before and after evaluation of a SBCC campaign. Data collection took place during 5 rounds of telephone interviews between June 2020 and April 2021 (Figure 1). During each round of data collection all eligible households were interviewed, and the respondent was asked questions about all household members. The enumerators conducted telephone interviews using a closed questionnaire designed using Open Data Kit. For the analysis presented in this paper, we used data from rounds 4 and 5, which were conducted in November 2020 and April 2021.

### 2.3. Design of the SBCC Campaign

The wave two ‘*i*MaskUp’ campaign was designed and led by the Inter-Agency Risk Communications and Community Engagement (RCCE) Task Force, funded by UNICEF, and implemented with partners to combat the second wave of COVID-19 in Somalia. The objective of the campaign was to promote three prevention behaviours (‘Hands, Face, Space’), with a focus on the use of homemade face masks, and increase the acceptability of COVID-19 vaccination.

The technical health promotion content was humanised using deontological messaging, and coordinated, high quality multi-media content was produced and made available under a national umbrella campaign. All content could be freely used by Government, UN agencies, NGO partners, and local community networks. Organisations were supported to deliver the content using two-way community engagement approaches. Work began in November 2020 with the drafting of a campaign strategy, and a more detailed campaign concept in December 2020. There was a level of resistance to working on COVID-19 in November and December 2020 given the high levels of stigma and life appearing to return to normal.

#### 2.3.1. Content Design and Production

The production of short, high-quality, video content was prioritised due to the availability of TV in urban areas and widespread access to social media more widely (particularly Facebook). A Somali filmmaker was commissioned to produce the videos and worked closely with one of the authors (MB). This was supplemented with audio content and graphic designs which were produced by a local media company. A key element of the campaign was the consistent use of the campaign slogans across different content. These were:

‘Hands, Face, Space’ (‘*Dhaq Gacmahaaga*, *Xiro Af-Xir*, *Ilaali Kala Fogaanshaha*’ in Somali) was borrowed from the Public Health England campaign. It was adopted for use in Somalia due to its simplicity and expected memorability in conveying key behaviour practices. ’I Mask Up’ (‘*Waxaan af-saab u xirtaa*’) used a deontological approach to remind people that prevention was about protecting others as much as themselves.

‘I Protect My Family’ (‘*Waxaan bad baadinayaa inta aan masuulka ka ahay*’) was adapted from an Islamic Hadith which guides Muslims to look after their own families and communities.

Content was pre-tested to ensure their language was accurate and accessible and it complied with local ethical norms.

#### 2.3.2. Influencers

Encouraging and working with influencers was an important part of the campaign strategy. These included political and religious leaders, who were encouraged to wear face masks, and the national football team, who were involved in a series of short films. These featured them wearing face masks while stating who they were wearing them for “I Mask Up from my grandmother”, etc. A well-known musician also appeared in the video series. Attempts to engage Somalia diaspora in the campaign were, however, less successful.

#### 2.3.3. Communication Channels

The campaign used a range of traditional and modern communication channels to try and reach a wide cross-section of different audiences. These included:

*Social media*— Somalis’ participation in social media, especially Facebook, and increasingly Instagram, meant this was a key space to share content and engage with Somalia’s youthful population who are increasingly active on this platform. Quote cards aligned to the campaign, 5 s video clip summaries of the longer videos, and Facebook profile photo frames, were created to generate engagement and discussion.

*Radio*—Radio was a likely trusted source of information. The RCCE drafted scripts for public service announcements (PSAs), and these were played on a wide network of national and state-wide commercial radio stations. The government also played the PSAs on Government Radio.

*Television*—Two of the short films produced (IMask Up and I Protect My Family) were aired on national TV channels with government endorsement and Ministry of Health logos added to the graphics at the end.

*Community mobilisers and Community Health Workers*—These frontline workers were key to reaching people face-to-face. While social distancing guidelines meant this had to be limited, household visits were done under the guidance of COVID-19 Standard Operating Procedures and a regularly updated frequently asked questions. Several actors, including UNICEF, used digital platforms to document their engagement and a live report of current rumours was tracked and shared with the RCCE and partner agencies.

*Mobile public address systems*—Agencies such as UNICEF, used mobile trucks to disseminate information. Radio quality audio content was created and put onto USB sticks for the local teams. Trucks were tasked to go to pre-mapped, community gathering spots, such as markets and mosques, and play the content and engage with the community on the issues raised. The audio content included music, drama, people’s science explanations of why the prevention methods were needed, and FAQs.

*Posters and leaflets*—The use of written content was minimised due to high levels of illiteracy in Somalia. However, printed billboards using the ‘Hands, Face, Space’ slogan were utilised by some State governments in locations outside airports or hospitals where there was frequent traffic and literacy levels were likely to be higher.

#### 2.3.4. Targeting Hard to Reach Groups

Anecdotal reports suggested that young men were largely ignoring advice on COVID-19. To reach this audience group an audio message which drew on Islamic Hadith’s was produced. These messages were recorded by the National Islamic Advisory Group (NIAG), and scripts shared with the Ministry of Religious Affairs. In addition, a separate audio script was produced that included a two-minute drama with two male protagonists discussing the increase in cases and prevention measures. The content was played on a network of mobile trucks with speakers that played the messages at listening stops in COVID-19 hotspot areas and in IDP sites. The inclusion of football players in the campaign content was also designed to reach this young male audience.

#### 2.3.5. Content Approval, Hosting, and Dissemination

All campaign content for the Wave 2 campaign was produced without UN logos and both with and without logos of the Federal Government of Somalia, to try and ensure that they could be used in different areas of Somalia by different actors. All video content was produced with multiple versions—one in English and others in different Somali languages. The complex political landscape of Somalia meant that content had to be produced and approved by three different Ministries of Health (Federal MOH, Puntland MOH and Somaliland MOH). For Somaliland, all content had to be re-produced separately using local actors and companies, and re-translated scripts. For Puntland, the scripts were tweaked to allow for the local dialect and voiced over. Following approval, all campaign content was uploaded to a UN managed, publicly accessible, web site and widely promoted to RCCE members and humanitarian organisations [27]. Further details of the design and implementation timeline for the SBCC campaign is given in Web Table S1.

### 2.4. Study Participants

**Figure 2:**
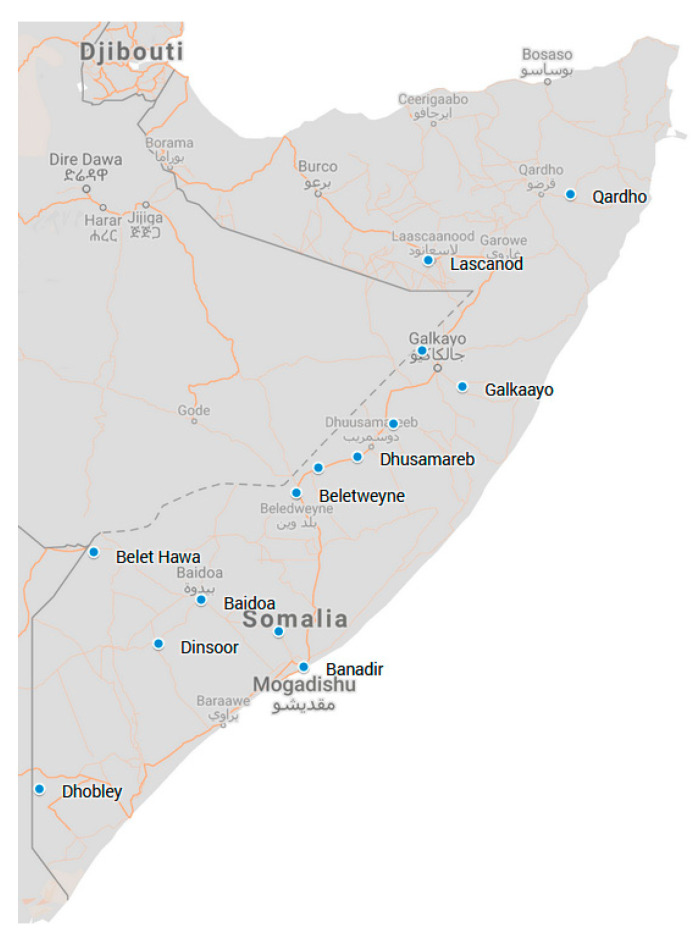
The distribution of participant households by district locations are shown with a blue dot.

Detailed methods have been described elsewhere [23]. In brief, the study participants were households enrolled in a long-term cash transfer programme (USD 20.00/household/month for two years). The programme was run by a consortium of NGOs and led by the Norwegian Refugee Council (NRC) [28] and served 43 communities across 10 regions of Somalia: Banadir, Bari, Bay, Galgadud, Gedo, Hiran, Lower Juba, Lower Shabelle, Mudug, and Sool. It reached 3048 households in total. Participant households included pastoralists, agro-pastoralists, IDPs, and urban residents. The households were selected by representatives of the community based on vulnerability criteria. The districts in Somalia where the respondents were sampled is shown in Figure 2.

### 2.5. Data Collection

Prior to the onset of the pandemic, telephone interviews were conducted periodically with a sample of beneficiaries for post-distribution monitoring purposes. With the outbreak of COVID-19, we adapted the approach; all household members were enumerated, and a longitudinal database was created to track mortality, attitudes to COVID-19 and vaccination, and behaviours associated with transmission risk.

From November 2020 until April 2021, a team of 15 enumerators, closely supported by consortium technical staff, periodically collected data by conducting telephone interviews with a household respondent. During the interview, data were entered into a second mobile phone running Open Data Kit Collect and subsequently uploaded to a server run by ONA. The telephone interviews had a median duration of 30 min, with some variability between different data collection rounds due to the inclusion or removal of questionnaire items. Consent for the interview was obtained verbally and recorded in the questionnaire.

Enumerators were selected based on previous experience in public health surveys and familiarity with conducting telephone interviews with vulnerable populations. An initial training session was held plus refresher trainings before each round of data collection. Real time data quality checks included the monitoring of interview duration, completeness, and non-response patterns.

### 2.6. Data Analysis

Data were downloaded from the ONA servers as .csv files and compiled and cleaned in Excel Power Query. Analysis was conducted using Stata v17 and graphs made in Excel. Calculation of a preventative behaviour score (PB-Score) was performed using scores based on contemporary public health advice issued by WHO. Use of face coverings, handwashing, and avoidance of hugging received a maximum score of 3, while avoiding hand shaking received a maximum score of 2. The total possible score ranged from 0 to 11 points (Table 1).

Mixed effects, multilevel models were used to test for associations between exposure to messages and behaviours and attitudes. District and community were included as random effects, and household livelihood, respondent age, and sex were included as fixed effects.

## 3. Results

The sample characteristics from each data collection round are shown in Table 2. The sample was taken from 45 communities in 20 districts. The target sample varied from round to round due to administrative changes to the beneficiary lists and an increase in the scope of the safety net intervention, which was scaled up in response to humanitarian needs caused by locust infestation and the COVID-19 pandemic. The response rate achieved for each round varied from 85% to 93%. Phones switched off, low batteries, or poor reception were anecdotally reported as the main reasons for non-response. The number of refusals was very low with less than 6 refusals reported per round.

The average duration of interviews varied by round due to the phase of data collection and adaptation of the questionnaires. During round 5, the mean duration of each interview was 36 min. The majority of respondents were female, and females comprised just over half of all household members. The age of the household respondent ranged from 16 to 95 years.

The trend in the suspected symptomatic COVID-19 infection rate is shown in Figure 1, along with the timing of the SBCC campaign. As described elsewhere, the suspected infection rate, determined using our syndromic case definition, was much greater due to the low levels of testing performed in Somalia [23]. The Wave 2 *i*MaskUp SBCC campaign was launched in February 2021 and lasted until the start of May.

The data collected in rounds 4 and 5 indicate there was a marked change in perceptions of the threat posed by COVID-19 between the end of the first wave and the start of the second. Table 3 shows that the proportion of households considering it to be a major threat increased from 46 to 70%. There was no difference in the perception threat by sex or age (*p* > 0.05). The perception that COVID-19 was only a threat to non-Muslims was reported by 6% of respondents at the end of wave 1 and did not change significantly.

Sixty-five percent of respondents reported hearing information or news on COVID-19 during the last 30 days at the end of wave 1, and this increased to 88% during wave 2. People received news and information from a wide variety of sources. Messages received by phone were reported by 9 out of 10 respondents that had received information, and this probably reflects the routine broadcasting of a pre-recorded information message by a national telecommunications company, Hormuud, to their subscribers each time they commenced a call. Radio broadcasts were the second most frequently reported source of information, followed by Community Health Workers or NGO workers, and religious leaders. Social media was reported by less than 1 in 5 but did increase significantly as wave 2 began.

Sources are ranked according to the reported level of trust in Table 4. Trust in the available sources of information was generally quite high with over 80% of respondents agreeing or strongly agreeing that they trusted information from religious leaders. Trust was lowest for social media and internet web pages, with about 30% of respondents trusting these sources.

Preventative behaviours increased markedly during wave 2 (Table 4), with a significant increase in the combined behavioural PB-Score. Wearing face masks every day increased by 16% and there was a large drop in those who never used them. Never using a face mask during wave 2 was higher in males by 20 percentage points (*p* = 0.001) and there was an increasing trend with age. Avoiding hand shaking every day increased, with half the respondents reporting this practice at during Wave 2. Women were more likely to avoid handshaking completely (61%) than men (41%) (*p* < 0.0001), but there was no significant difference by age.

Hugging was a relatively rare social practice with only 13.5% of respondents having hugged anyone outside of their household in the last week. This fell further during the start of the second wave with 9 out of ten people avoiding it completely. Women (91%) were more likely than men (79%) to avoid hugging completely (*p* = 0.002), but there was no difference by age. Overall, preventative behaviours, measured using the PB-Score, increased significantly during the second wave, the PB-Score being higher in women than men (8.6 vs. 7.4, *p* < 0.0001).

Data on vaccine acceptance and reasons for refusal are given in Web Figures S1 and S2. Seventy percent of respondents stated that they would accept a vaccination against COVID-19 if they were offered it, with a similar proportion recommending it for an elderly relative. The proportion accepting vaccination decreased with age, falling from 77% in those under 25 years to 65% in those over 50 years (*p* = 0.009) and was higher in males than females (75.5% vs. 67.0%, *p* = 0.015). The main reasons given for reusing vaccination were lack of information, concerns about side effects or safety, and the notion that it is Allah who will decide their fate.

To assess the extent of exposure to the SBCC campaign, we asked in round 5 whether they had heard the specific slogans used by the campaign. A high proportion of respondents reported having heard the three campaign slogans: ‘*Hands face space*’, ‘*I mask up*’, and ‘*I protect my family*’ (Table 5). Half of the households reported that someone in the household had made their own face covering, a strategy recommended in the campaign. Awareness of SBCC campaign slogans was not different by sex (*p* > 0.05) but was higher for all slogans in younger age group (*p* < 0.05). It also differed by livelihood, with lower exposure to all 3 slogans reported by those with pastoral livelihoods, compared to agricultural or urban livelihoods (*p* < 0.05).

To assess if exposure to the SBCC campaign had contributed to the changes in attitudes and behaviours, we constructed multi-level regression models. This analysis revealed that some behaviours were associated with exposure to particular SBCC slogans. The three slogans were used in a variety of formats and in different combinations. Slogans 1 and 2 were used in materials aimed at increasing social distancing behaviours. Slogan 2 and slogan 3 were used in materials aimed, in particular, at increasing use of face masks and reducing vaccine hesitancy [27]. As shown in Table 6, exposure to slogan 2 was associated with increased use of face masks and exposure to slogans 2 and 3 with increased vaccine acceptance. No slogan exposures were associated with changes in hand shaking or hugging, or hand washing frequency.

## 4. Discussion

This study shows how risk perception, behaviours, and attitudes changed between successive waves of the COVID-19 pandemic in Somalia. We also demonstrated the association of a novel, national, SBCC campaign with beneficial changes in behaviour and vaccine acceptance. This study took place during the early phases of the pandemic, while there was still considerable uncertainty about the virulence of the virus, the emergence of different variants, and the population impact that would occur in low-income countries in Africa. It also took place before the emergence of the more transmissible Omicron variant of SARS-CoV-2 [29].

Our analysis showed that attitudes and behaviours changed markedly in between the end of wave 1 and the beginning of wave 2. Changes in the public awareness of COVID-19 are likely to have been driven by a range of factors in addition to the SBCC campaign. These included high profile events, such as the death of a former President of Somalia from COVID-19 in March 2021, as well as general media coverage [30]. The increase in the perceived level of threat was associated with an increasing adherence to social distancing, hand washing, and mask wearing precautions. Women were more likely to adopt precautionary behaviours as reflected by a higher PB-Score.

The SBCC campaign was designed to use a people-centred, deontological approach, that minimised use of top-down messaging, and the creation of content followed these principles. This content was supported by on the ground engagement strategies that aimed to involve people in conversation and supported listening, and encourage them to take precautionary measures. There was a high level of awareness of the SBCC campaign messages and exposure to specific messages was associated with increases in mask wearing but no other NPI behaviours.

Vaccine acceptance was found to be quite high, and at a similar level reported during the first year of the pandemic for the general population in South Africa and other African countries [5,9]. Acceptance was higher in males but decreased in both sexes with age. These finding agree, in general, with other studies conducted in Africa [14,15,16,17]. Exposure to SBCC messages was associated with an increased level of acceptance. These associations provide evidence that the campaign contributed significantly to beneficial changes in attitudes and practices and that it had an additive effect, layering on top of increased public concern about the second wave and the perceived threat level. The importance of adopting non-coercive public policies and actions to encourage vaccine acceptance, as well as factors such as public trust and community involvement has been emphasised [31]. Given the ongoing conflict and fragile nature of the political situation in Somalia, the apparent success of the campaign is remarkable.

The study used a convenience sample based on the geographic and socio-economic distribution of the pre-existing BRCiS cash transfer programme beneficiaries. While this did not allow for the design of a population representative sample, it did mean that data collection could begin rapidly during the pandemic. The targeting criteria used to select the beneficiaries are likely to have resulted in the recruitment of an economically and socially vulnerable population. Thus, our findings need to be interpreted with that in mind.

The collection of longitudinal data provided the opportunity to assess changes in the same population sample over time and at different stages of the pandemic. Telephone interviews allowed access but may have led to different types of bias compared to face-to-face interviews. The questionnaire we used was designed specifically for this study and it was not possible to conduct formative studies to confirm its validity. The study design also meant that the study participants had been repeatedly interviewed, over five data collection rounds, since the onset of the pandemic. During each interview, they were provided with information about COVID-19, so the awareness level may well have been higher than in the general population. However, it is important to note that the specific campaign slogans that were investigated in this paper were not used in the information routinely provided after each data collection round.

The SBCC campaign used multiple channels and materials to reach its target audience. Due to the many audio–visual materials that were produced and distributed via different channels, it was not feasible to ask respondents if they were aware of each of them. Instead, we asked if they were aware of three key campaign slogans and used this to estimate their exposure to the different materials and approaches used in the campaign.

We conclude that the use of telephone interviews to collect data on attitudes and behaviours is a useful approach in outbreaks where face-to-face interviews are undesirable. The SBCC campaign achieved widespread penetration and was associated with important changes in preventative behaviours and attitudes to vaccination. The use of a people centred, deontological, multimedia approach and a wide variety of channels appear to have contributed to the success of the campaign. Experience from this work should help inform future SBCC campaigns in Somalia and elsewhere.

## Figures and Tables

**Table 1:** Calculation of the prevention behavior score (PB-Score).

Behavior Frequency	Scores		
Days/Week	Face Mask Use	Social Hugging	Social Handshaking
Everyday	3	0	0
Most of the days	2	1	0
Some days	1	2	1
Never	0	3	2
**Times/day**	**Handwashing**		
0	0		
1–3	1		
4–6	2		
>6	3		

**Table 2:** Participant characteristics ^1^.

Measurement Period	Round 4		Round 5	
Data Collection	30 November–20 December 2020		27 March–18 April 2021	
Households interviewed	884		1403	
Household size (range, SD)	8.4	(1–22, 3.0)	8.4	(1–21, 3.1)
Respondent sex, % female (95% CI)	62.9	(59.7, 66.0)	66.5	(64.0, 68.9)
Respondent age, years (range, SD)	43.1	(13–95, 16.1)	43.3	(16–95, 15.9)
Livelihood type (%)				
Agricultural	16.5	(14.2, 19.1)	23.1	(21.0, 25.4)
Pastoral	31.8	(28.8, 34.9)	26.5	(24.3, 28.9)
Agropastoral	5.4	(4.1, 7.1)	5.7	(4.6, 7.0)
Riverine	0.0	-	0.9	(0.5, 1.6)
Urban	46.3	(43.0, 49.6)	43.8	(41.2, 46.4)
HH members with symptomatic COVID-19	12		81	
Period prevalence of symptomatic COVID-19	0.2%		0.7%	
Symptomatic COVID-19 infection rate (cases/1,000,000/day) ^2^	34		78	

^1^ Data on behaviours were obtained only from the household respondent while all household members were included in the calculation of symptomatic COVID-19. ^2^ In round 4, the recall period was the number of days since the previous interview. In round 5, it was the first 3 months of 2021.

**Table 3:** Changes in perceptions of COVID-19 and sources of information between end of wave 1 and beginning of wave 2 ^1^.

Data Collection Round	Round 4		Round 5		Δ	(95% CI)	*p*-Value
N	884		1403				
How much of a threat, if any, is COVID-19 to your health and to the health of your family? %, (95%CI)							
Not a threat	7.9	(4.4, 13.7)	5.8	(2.8, 11.4)	−2.1	(−7.3, 3.0)	0.462
A minor threat	45.9	(33.0, 59.4)	24.7	(15.7, 36.6)	−21.3	(−39.8, −2.7)	**0.023**
A major threat	46.2	(31.4, 61.6)	69.6	(56.9, 79.8)	23.4	(2.5, 44.3)	**0.021**
Do respondents who believed that COVID-19 is a disease that can affect only non-Muslims? %, (95% CI)	6.2	(3.8, 10.1)	9.5	(5.9, 14.8)	3.3	(−2.3, 8.8)	0.241
Proportion of respondents who had received news or information about COVID-19 in last 30 days. %, (95% CI)	64.7	(55.3, 73.1)	87.9	(75.4, 94.5)	23.2	(10.8, 35.6)	**<0.0001**
Sources of information for respondents who had received news or information in last 30 days %, (95% CI) ^1^							
Religious Leaders/Mosque	15.9	(8.3, 28.2)	36.8	(24.5, 51.1)	20.9	(7.6, 34.2)	**0.003**
Phone	67.3	(53.3, 78.8)	90.6	(76.9, 96.5)	23.3	(7.4, 39.2)	**0.005**
Community health workers/NGO workers	33.6	(21.9, 47.6)	38.8	(28.3, 50.3)	5.2	(−9.1, 19.5)	0.467
Community Resilience Committees	27.6	(17.6, 40.5)	30.7	(21.8, 41.2)	3.0	(−11.8, 17.9)	0.682
Government sources	10.0	(5.2, 18.3)	19.4	(12.5, 28.9)	9.4	(−0.9, 19.7)	0.072
Radio	71.3	(56.3, 82.8)	72.1	(59.3, 82.1)	0.8	(−17.0, 18.6)	0.931
Women’s group	20.8	(12.0, 33.6)	14.4	(8.2, 23.9)	−6.4	(−17.8, 4.9)	0.256
Social media or web sites	8.0	(4.0, 15.5)	17.0	(11.6, 24.4)	9.0	(0.4, 17.5)	**0.040**
Other	38.5	(25.4, 53.4)	25.2	(18.4, 33.5)	−13.2	(−29.3, 2.8)	0.104

^1^ Sources are ranked by the level of trust reported in round 5.

**Table 4:** Change in prevention behaviours reported between end of wave 1 and beginning of wave 2.

Data Collection Round	Round 4		Round 5		Δ	(95% CI)	*p*-Value
N	884		1403				
Number of times *hands washed* per day	7.7	(7.0, 8.4)	8.2	(7.6, 8.9)	0.5	(−0.3, 1.4)	0.228
Use of *face masks* when outside of their home (%):							
Everyday	4.1	(2.0, 8.6)	20.0	(12.7, 30.1)	15.8	(7.1, 24.6)	**0.001**
Most of the days	9.0	(5.4, 13.8)	26.3	(20.6, 33.0)	17.6	(12.0, 23.2)	**<0.0001**
Some days	27.5	(18.4, 39.0)	18.3	(14.8, 22.4)	−9.2	(−19.9, 1.6)	0.092
Never	59.6	(14.8, 22.4)	35.4	(24.9, 47.4)	−24.3	(−36.5, −12.1)	**<0.0001**
*Shaking hands* with people outside of their household (%):							
Everyday	7.2	(4.3, 11.9)	3.4	(1.5, 7.5)	−3.9	(−8.8, 1.0)	0.114
Most of the days	19.2	(14.2, 25.5)	9.8	(6.8, 13.8)	−9.5	(−15.8, −3.2)	**0.004**
Some days	42.5	(34.6, 50.9)	32.6	(25.2, 41.1)	−9.9	(−19.4, −0.4)	**0.042**
Never	31.0	(22.1, 41.5)	54.2	(43.7, 64.4)	23.2	(9.8, 36.7)	**0.001**
*Hugging* someone from outside of their household (%):							
Everyday	0.2	(0.1, 0.9)	1.8	(0.5, 6.3)	1.6	(−0.8, 3.9)	0.185
Most of the days	5.9	(3.6, 9.6)	2.5	(1.2, 5.3)	−3.4	(−6.9, 0.2)	0.061
Some days	24.5	(18.5, 31.8)	9.2	(5.7, 14.4)	−15.4	(−22.6, −8.1)	**<0.0001**
Never	69.3	(60.0, 77.3)	86.5	(78.2, 92.0)	17.2	(6.2, 28.2)	**0.003**
Combined Prevention Score (PB-Score) (mean, 95% CI)	6.9	(6.6, 7.3)	8.2	(7.7, 8.7)	1.3	(0.7, 1.8)	**<0.0001**

**Table 5:** Exposure to the Wave 2 SBCC Campaign in round 5.

Campaign Component (N = 1403)	%	(95% CI)
Has heard the phrase ‘*Hands face space*’ (slogan 1)	87.5	(80.4, 92.2)
Has heard the phrase ‘*I mask up*’ (slogan 2)	71.3	(63.5, 78.3)
Has heard the phrase ‘*I protect my family*’ (slogan 3)	67.8	(59.9, 74.9)
Someone in household has made their own face mask	52.9	(39.9, 65.5)

**Table 6:** Multilevel ordered or binary logistic regression models of the association of SBCC message awareness with COVID-Prevention behaviours and vaccine acceptance at the beginning of wave 2 ^1^.

SBCC Campaign Slogan	Target Behaviour/Attitude	Exposure to Slogan	Mean Behaviour Scoreor Adjusted Attitude Prevalence (%)	SD or 95% CI	Odds Ratio	(95% CI)	*p*-Value
(1) ‘*Hands face space*’	Social distancing: hugging ^2^	−	2.85	0.47	Ref.	-	-
		+	2.79	0.57	0.75	(0.18, 3.14)	0.690
	Social distancing: hand shaking ^2^	−	2.63	0.67	Ref.	-	-
		+	2.34	0.80	0.41	(0.11, 1.56)	0.192
	Handwashing: daily frequency ^2^	−	2.40	0.72	Ref.	-	-
		+	2.71	0.53	0.76	(0.35, 1.66)	0.488
	Use of face covering ^2^	−	1.09	0.95	Ref.	-	-
		+	1.34	1.17	1.37	(0.63, 2.95)	0.426
(2) ‘*I mask up*’	Social distancing: hugging ^2^	−	2.86	0.47	Ref.	-	-
		+	2.78	0.59	0.93	(0.40, 2.18)	0.872
	Social distancing: hand shaking ^2^	−	2.51	0.75	Ref.	-	-
		+	2.33	0.81	0.88	(0.52, 1.49)	0.627
	Handwashing: daily frequency ^2^	−	2.59	0.65	Ref.	-	-
		+	2.71	0.53	0.90	(0.42, 1.92)	0.781
	Use of face covering ^2^	−	0.94	1.00	Ref.	-	-
		+	1.46	1.46	2.31	(1.52, 3.51)	**<0.0001**
	Vaccination acceptance ^3^	−	58.0%	0.53, 0.63	Ref.	-	-
		+	76.4%	0.69, 0.84	2.53	(1.58, 4.06)	**<0.0001**
(3) ‘*I protect those I am responsible for*’	Vaccination acceptance ^3^	−	60.0%	53.5, 66.4	Ref.	-	-
		+	76.7%	70.2, 83.2	2.36	(1.60, 3.50)	**<0.0001**

^1^ The models were adjusted for the fixed effects of respondent age, sex, and the household’s livelihood. District and community locations were included as random effects. ^2^ Analysed using ordered logistic regression. ^3^ Analysed using binary logistic regression.

## Data Availability

The data presented in this study are available on request from the corresponding author. The data are not publicly available due to concerns about confidentiality and security in Somalia.

## Supplementary Materials

The following supporting information can be downloaded at https://www.mdpi.com/article/10.3390/vaccines11050972/s1. Web Table S1: Timeline and key milestones for the design and implementation of the Wave 2 iMaskUp SBBC campaign1. Web Figure S1: Data on vaccine acceptance and reasons for refusal (1). Web Figure S2: Data on vaccine acceptance and reasons for refusal (2).
