# Supplementary material for: COVID-19 Prevention Behaviours and Vaccine Acceptability, and Their Association with a Behaviour Change Campaign in Somalia: Analysis of a Longitudinal Cohort
Source: Vaccines (Basel). 2023 May 11;11(5):972. doi: 10.3390/vaccines11050972 (PMC10220919; doi:10.3390/vaccines11050972)
Supplement: Supplementary file 1 [file vaccines-11-00972-s001.zip › Web Table S1.pdf]

Web Table S1 Timeline and key milestones for the design and implementation of the Wave 2 iMaskUp SBBC campaign[1]

**Phase**	**Date**	**Activity**
Review	Nov-Dec 2020	Informal assessment of the Wave One campaign and Wave Two strategy design.
Design	Dec 2020-Jan 2021	Wave two campaign design and preparations (drafting messages and content and testing language).[1]
Content creation	Feb 2021	1. Campaign slogan design with local graphics company ‘Hands. Face. Space’ [2] (for social media, strategically placed billboards / banners e.g. in hospitals, and for community engagement).<br>2. Commissioning of video production for four short ‘hero’ films<br>• Hands-Face-Space<br>• I Mask Up<br>• How to stitch a face mask<br>• I Protect My Family – vaccine uptake film.<br>3. Commissioning and drafting a script for Hiro and Hirshi (existing well-known Somalia Sesame Street style puppets) video production for children.<br>4. Commissioning and drafting scripts for three simple explainer animations, that featured two cartoon characters called Leila and Adaan, on how COVID-19 is spread and why the prevention measures work.<br>5. Creation of audio and online content for Imams and congregations, which were later shared through the National Islamic Advisory Group.
Campaign launch	Feb 2021	1. Launch of national and local radio Public Service Announcements (PSA) for ‘Hands, Face, Space’ and ‘I Mask Up’.<br>2. Launch of PSA for ‘I Protect My Family’ and vaccine uptake.
Campaign implementation	Mar 2021	1. March 6th: ‘I Mask Up’ video launched together with mask making instructions flyer that was distributed through UN and NGO networks. (Within days these had millions of views on Facebook)<br>2. March 11th: ‘Hands Face Space’ video launched.<br>3. March 21st: ‘How to make a face mask at home’ video launched.<br>4. March 30th: ‘I protect My Family’ video launched on vaccine awareness to encourage acceptance.<br>5. Audio content scripted, produced and shared on USB sticks for roving trucks to play on speakers at dedicated listening stops. Content included music from popular Somali singer Aar Manta, an original three-minute drama, and people’s science explanations of why prevention methods were effective.<br>6. Social media quote cards launched with campaign messaging and logos, linked to ‘Hands Face Space’, and ‘IMaskUp’ slogans.<br>7. Facebook profile frames created and launched for vaccine uptake “I protect my Family / I took my COVID-19 vaccine.”
	Apr 2021	1. April 13th: Video with national football team and female activists released<br>2. April 21st: Series of animations with two characters Layla and Aadan launched.<br>3. Government Ministers photographed taking the vaccine and adoption of social media profile photo frames designed by the campaign.

[1] An important policy development occurred on1st March 2021 when the Federal Government in Mogadishu made face masks mandatory in public places and social gatherings.

[1] For the Wave Two iMaskUp *draft* campaign design concept and initial ideas see: https://www.humanitarianresponse.info/sites/www.humanitarianresponse.info/files/documents/files/masks_campaign.pdf

[2] The Hands-Face-Space slogan was the campaign slogan adopted by Public Health England for its COVID-19 prevention message. The first public health video for Somalia leant on the Public Health England TV advert that was widely aired in the UK https://www.youtube.com/watch?v=jr09ByDYuq4. The Somali versions used local actors and settings to mirror this content and a version was created for a national audience, as well as versions for Puntland and Somaliland audiences.
