# Supplementary figures and images for: COVID-19 Prevention Behaviours and Vaccine Acceptability, and Their Association with a Behaviour Change Campaign in Somalia: Analysis of a Longitudinal Cohort
Source: Vaccines (Basel). 2023 May 11;11(5):972. doi: 10.3390/vaccines11050972 (PMC10220919; doi:10.3390/vaccines11050972)

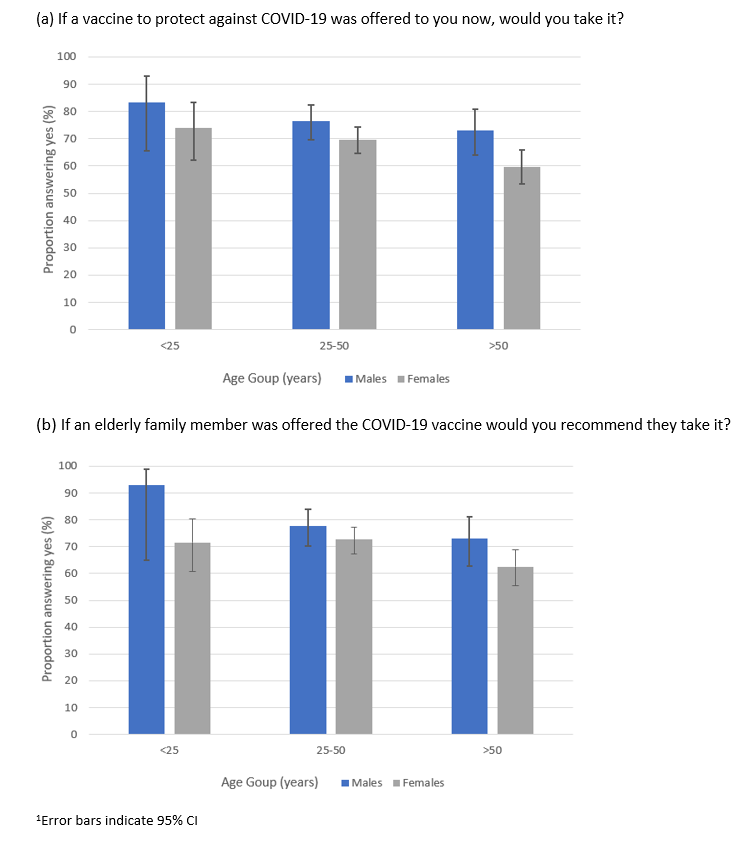

Supplement: Supplementary file 1 [file vaccines-11-00972-s001.zip › Web Figure S1.png]

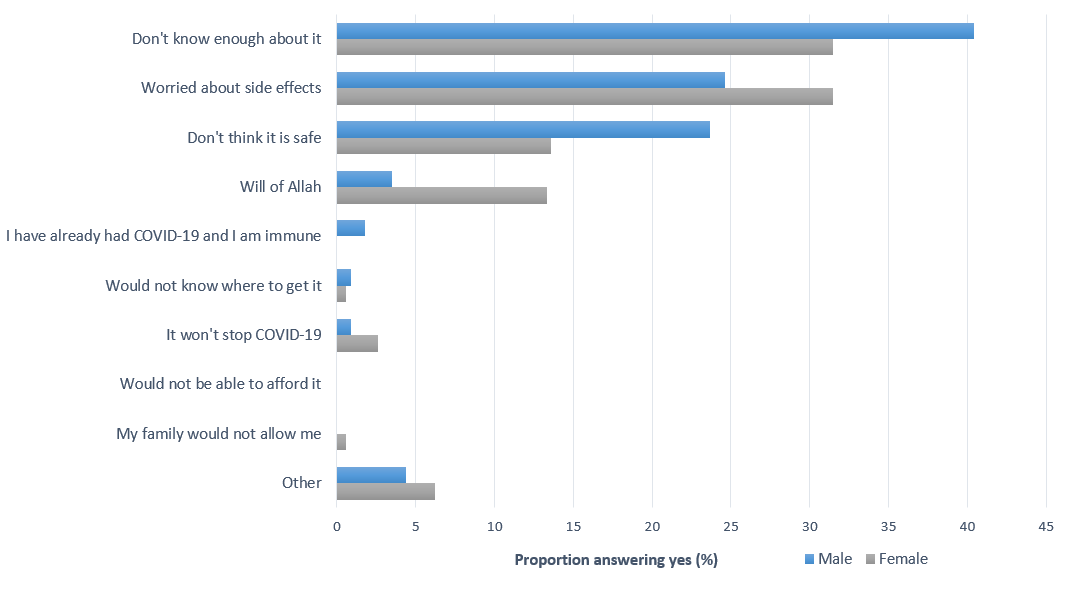

Supplement: Supplementary file 1 [file vaccines-11-00972-s001.zip › Web Figure S2.png]
